# Deep learning-based spatio-temporal fusion for high-fidelity ultra-high-speed X-ray radiography

**DOI:** 10.1107/S1600577525000323

**Published:** 2025-02-12

**Authors:** Songyuan Tang, Tekin Bicer, Tao Sun, Kamel Fezzaa, Samuel J. Clark

**Affiliations:** a Advanced Photon Source Argonne National Laboratory Lemont IL60439 USA; b Data Science and Learning Division Argonne National Laboratory Lemont IL60439 USA; c Department of Mechanical Engineering Northwestern University Evanston IL60208 USA; Australian Synchrotron, Australia

**Keywords:** X-ray imaging, deep learning, high-speed imaging, spatio-temporal fusion, full-field X-ray radiography

## Abstract

Full-field ultra-high-speed (UHS) X-ray imaging experiments have been well established to characterize various processes and phenomena. However, the potential of UHS experiments through the joint acquisition of X-ray videos with distinct configurations has not been fully exploited. In this paper, we investigate the use of a deep learning-based spatio-temporal fusion (STF) framework to fuse two complementary sequences of X-ray images and reconstruct the target image sequence with high spatial resolution, high frame rate and high fidelity. We applied a transfer learning strategy to train the model and compared the peak signal-to-noise ratio (PSNR), average absolute difference (AAD) and structural similarity (SSIM) of the proposed framework on two independent X-ray data sets with those obtained from a baseline deep learning model, a Bayesian fusion framework and the bicubic interpolation method. The proposed framework outperformed the other methods with various configurations of the input frame separations and image noise levels. With three subsequent images from the low-resolution (LR) sequence of a four times lower spatial resolution and another two images from the high-resolution (HR) sequence of a 20 times lower frame rate, the proposed approach achieved average PSNRs of 37.57 dB and 35.15 dB, respectively. When coupled with the appropriate combination of high-speed cameras, the proposed approach will enhance the performance and therefore the scientific value of UHS X-ray imaging experiments.

## Abstract 2

A deep learning-based algorithm is developed and evaluated that demonstrates the potential to reconstruct simultaneously high-resolution high-frame-rate X-ray image sequences with high fidelity through spatio-temporal fusion. Experimental evaluation shows that the method can significantly improve the accuracy of the reconstruction, achieving an average peak signal-to-noise ratio (PSNR) of more than 35 dB on two representative X-ray image sequences with input data streams of four times lower spatial resolution and 20 times lower frame rate, respectively.

## 1. Introduction

Ultra-high-speed (UHS) cameras typically refer to cameras that record videos at a frame rate of above 1 MHz and exposure times of less than 1 µs (Tochigi *et al.*, 2012 ▸; Sugawa, 2013 ▸). UHS cameras have been a key characterization tool utilized by a number of scientific user communities to investigate physical phenomena such as combustion, materials fracture, fluid dynamics and electric discharges (Miyauchi *et al.*, 2014 ▸; Manin *et al.*, 2018 ▸). As modern sensor technologies continue to improve, UHS cameras operating at higher frame rates become commercially available (Miyauchi *et al.*, 2014 ▸; Nguyen *et al.*, 2016 ▸; Etoh *et al.*, 2017 ▸).

In conventional full-field X-ray radiography, the concept of an indirect detector is a common experimental design choice which involves a scintillator that first converts the X-rays to visible light and a camera lens system that subsequently collects the light to form the X-ray images (Bonse & Busch, 1996 ▸; Hartmann *et al.*, 1975 ▸; Koch, 1994 ▸). Through the use of commercial cameras, indirect detectors greatly improve the flexibility of an X-ray experiment, leading to the development of customized instruments to meet the specific needs of myriad applications, such as additive manufacturing (and some other impactful use cases) (Douissard *et al.*, 2012 ▸). When a high-speed camera is used in such experiments, however, a trade off generally exists between its spatial resolution and its temporal resolution (Li *et al.*, 2022 ▸). In order to observe highly dynamic features at extremely fine temporal and spatial scales, imaging systems require frame rates and spatial resolutions that are not currently available. In other words, we face scientific demand for the highest acquisition frequency, largest field-of-view (FOV) and highest spatial resolution simultaneously, an experimental trilemma necessitating compromise for each experiment. As a result, in an experiment that normally utilizes a single high-speed camera implementation of different imaging settings, it is commonly necessary to repeat the same experiment multiple times to deploy the corresponding configurations. In the past, several multiplex setups consisting of more than one high-speed camera have also been proposed to image the same experimental phenomena with various detector configurations (Luo *et al.*, 2012 ▸; Ramos *et al.*, 2014 ▸; Escauriza *et al.*, 2020 ▸). So far, there has been no study within the area of full-field UHS X-ray imaging, and only limited studies in other related areas, to fuse multiple high-speed acquisitions to aggregate the distinct advantages of their underlying configurations. An exemplar configuration available on the 32-ID high-speed imaging beamline (Parab *et al.*, 2018 ▸) at the Advanced Photon Source (APS) would be to use simultaneously a UHS HPV-X2 (Shimadzu Corp., Japan) camera and an HS TMX7510 (Vision Research Inc., USA) camera, which at full resolution have maximum frame rates of 5 MHz (400 × 250 pixels) and 76 kHz (1280 × 800 pixels), respectively.

Beyond X-ray imaging, Kornienko *et al.* (2022 ▸) developed a method to illuminate the object with three temporally resolvable nanosecond laser pulses during one single camera exposure and unmix the resulting image signals at the post-processing time (Kornienko *et al.*, 2022 ▸). This method, termed ‘frequency recognition algorithm for multiple exposures’ (FRAME), allowed efficient recording of complex processes with motion dynamics manifested on multiple time scales. He *et al.* (2024 ▸) designed a multi-modal acquisition framework to image ultrafast phenomena with high fidelity. In their work, three imaging models, namely compressed ultrafast photography (CUP), transient imaging at a relatively lower frame rate and spatio-temporal integration, were implemented within one single acquisition and fused with an untrained neural network (UNN). The reconstructed transient image sequence had improved frame rates, with demonstrated higher peak signal-to-noise ratio (PSNR) and structural similarity (SSIM) than prior methods.

To build general-purpose video processing software, various forms of object motion need to be properly handled (Xue *et al.*, 2019 ▸). In particular, for the task of image restoration, the availability of neighboring frames, including those from co-registered imaging devices, could significantly extend the performance limit that can be achieved with a single image. One technique to restore low-quality video frames is called feature alignment. Broadly speaking, feature alignment methods can be categorized as explicit or implicit. Explicit methods first estimate and then compensate for motion fields between neighboring video frames to achieve alignment of corresponding features (Werlberger *et al.*, 2011 ▸; Fransens *et al.*, 2007 ▸; Caballero *et al.*, 2017 ▸). Recent work on explicit feature alignment has concentrated on the use of deep learning to improve the accuracy of the estimated motion fields and better adapt to specific vision tasks (Wang *et al.*, 2020 ▸). Due to the powerful feature extraction capability of deep neural networks, and compounded with the efficient development of deformable convolutions (Dai *et al.*, 2017 ▸; Zhu *et al.*, 2019 ▸), implicit feature alignment has recently gained great momentum. The deformable convolution was originally designed to improve a neural network’s adaptability in modeling varying object shapes and has demonstrated great success in applications such as object detection and semantic segmentation (Dai *et al.*, 2017 ▸). Later, Tian *et al.* (2020 ▸) proposed a novel deep neural network, termed a temporally deformable alignment network (TDAN), for video super-resolution. More specifically, those authors extended the use of deformable convolutions to the dynamic prediction of offsets and alignment of deep features between temporally neighboring image frames. Wang *et al.* (2019 ▸) further extended the deformable convolution module in TDAN to a hierarchical architecture to tackle larger object motions in videos of more dynamic scenes.

Even though accurate and reproducible video fusion software has been developed, there has still been no uptake in routine UHS X-ray radiography experiments. The availability of such a capability could fundamentally transform current research activities to enable the discovery of new phenomena of scientific value, while simultaneously providing simplified user-friendly experimental workflows. In other research areas such as communication networks and remote sensing, spatio-temporal image fusion technologies have received a great amount of interest, with emerging computational model architectures, data formats and machine learning algorithms demonstrating competitive performance (Hong *et al.*, 2020 ▸; Lu *et al.*, 2023 ▸; Chen *et al.*, 2024 ▸; Xiao *et al.*, 2024 ▸). Knowledge obtained in these developments could in turn be leveraged to improve UHS X-ray video fusion and reconstruction.

In this paper, we demonstrate the technical feasibility of fusing two sequences of high-speed X-ray images using a deep learning-based approach, with the intent of understanding the performance and limitations in reconstructing a single sequence of X-ray images with high spatial resolution and high frame rate. More specifically, we reorganized a video restoration framework with enhanced deformable convolution (EDVR) that fused a set of temporally consecutive low-resolution (LR) image frames, so it could integrate details from additional high-resolution (HR) input image frames to predict a target HR image frame with improved fidelity. This new model architecture aimed at spatio-temporal fusion, termed EDVR-STF, was pre-trained on image restoration benchmarks and subsequently fine tuned on dedicated X-ray image data sets for transfer learning. Performance was benchmarked against three existing approaches for image sequence restoration. In addition, we identified a new set of performance metrics, termed the ‘backward attention score’ and ‘forward attention score’, in order to interpret the effectiveness of the proposed method and give insight into the utilization of the input HR frames, in the absence of the actual HR frames as the ground truth.

## 2. Methods and numerical experiments

The model architecture and model training were based on the *BasicSR* package (Wang *et al.*, 2022 ▸). More details can be found in Section S1 of the supporting information. The training data were obtained from high-speed X-ray imaging experiments conducted at the Argonne National Laboratory APS on beamline 32-ID-B. The X-ray beam used for imaging was a pseudo-pink beam with a first harmonic energy of ∼24.5 keV and an energy bandwidth of ∼7% generated with a 1.8 cm short period undulator with a gap of typically 12.5 mm and a sample-to-detector distance of ∼400 mm. A 100 µm thick LuAG:Ce scintillator was used to convert the X-rays to visible light. The camera used was a Photron Fastcam SA-Z 2100K operating at a frame rate of 50 kHz, with an exposure time of 1 µs and a spatial resolution of 2 µm pixel^−1^.

**Figure 1:**
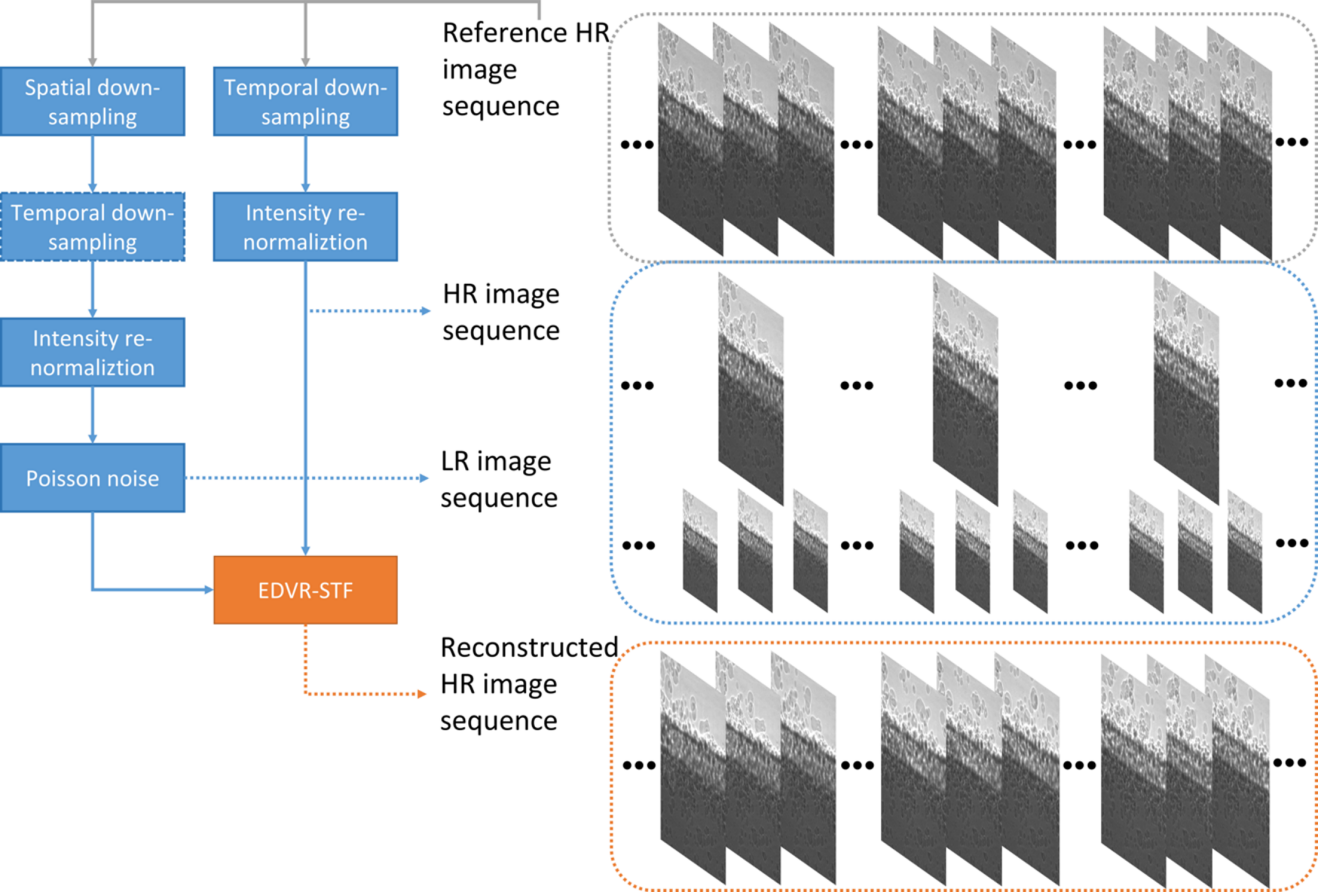
Illustration of the numerical experiment workflow.

Two independent X-ray videos for applications of additive manufacturing and friction stir welding were used to test the performance of the proposed image reconstruction algorithm. Both videos were acquired using the same Photron camera as used in the acquisition of the training data, operated at frame rates of 50 kHz and 20 kHz, respectively, on the 32-ID beamline of the APS (Ren *et al.*, 2023 ▸; Agiwal *et al.*, 2022 ▸). For each type of video, the pixel values were normalized, and spatial and temporal sub-samples were obtained at varying sampling rates to create the LR (and with ultra-high speed) image sequence and the HR (and with high speed) image sequence, respectively. In the case of the LR image sequence, Poisson noise of varying magnitudes was also simulated by varying the blank scan factor in a dedicated image degradation model (Wu *et al.*, 2020 ▸). For more details refer to Section S1.3 of the supporting information. Model inference was thus performed by iterating through each LR frame as the reference LR frame of the input, pairing it with another two LR frames before and after it at the designated frame separation and with two nearest HR frames before and after it, from the designated down-sampled frame sequences, respectively, and outputting the reconstructed HR frame at the same time as the reference LR frame. Fig. 1 ▸ illustrates the experimental configuration.

In addition to the proposed EDVR-STF reconstruction model, the same testing data were also used to evaluate the bicubic interpolation, a Bayesian image fusion framework previously developed for geospatial applications (Xue *et al.*, 2017 ▸), and the baseline EDVR reconstruction model for video super-resolution (Wang *et al.*, 2019 ▸). For more details refer to Section S1.4 of the supporting information. To evaluate the candidate reconstruction algorithms, a total of three performance metrics were considered: the peak signal-to-noise ratio (PSNR) (Wang *et al.*, 2019 ▸), the average absolute difference (AAD) (Zhang *et al.*, 2021 ▸) (range is 0–255) and the structural similarity (SSIM) index (Wang *et al.*, 2004 ▸). In the remainder of this paper, PSNR evaluated on the LR images with Poisson noise (relative to the LR images directly down-sampled from the original HR images) will be referred to as the ‘LR image PSNR’, to be distinguished from that evaluated on the reconstructed HR images (relative to the original HR images). In addition to these reference-based quality assessment metrics, we also analyze the contribution of each of the input HR images to the reconstruction of the target HR image. More details are given in Section S1.5 of the supporting information.

## 3. Results

**Figure 2:**
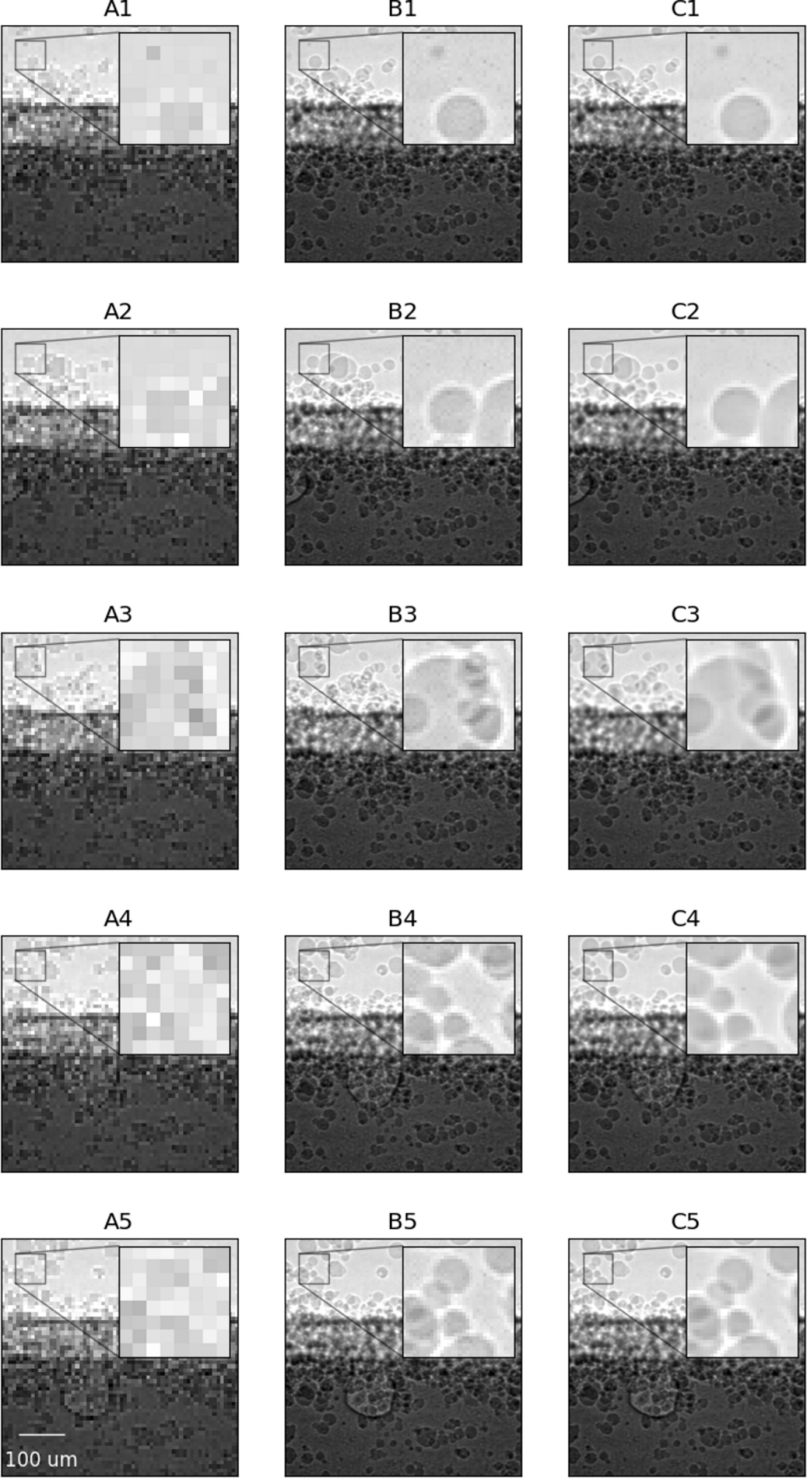
Selected image sequences of the LR images (column A), HR images (column B) and HR images reconstructed using EDVR-STF (column C) from Case 1. For each image sequence, images are shown from frames *i* − 10 (row 1), *i* − 9 (row 2), *i* (row 3), *i* + 9 (row 4) and *i* + 10 (row 5). To reconstruct HR images in the frame range [*i* − 10, *i* + 10], only HR images from frames *i* − 10 and *i* + 10 were used. No Poisson noise was generated for the testing data.

**Figure 3:**
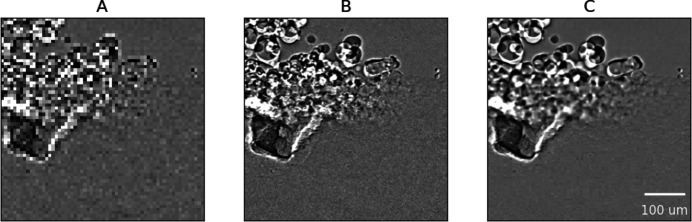
Selected (A) LR image, (B) HR image and (C) HR image reconstructed using EDVR-STF from row 3 of Fig. 2, divided, on the basis of the pixel value, by the same from their previous frame.

**Figure 4:**
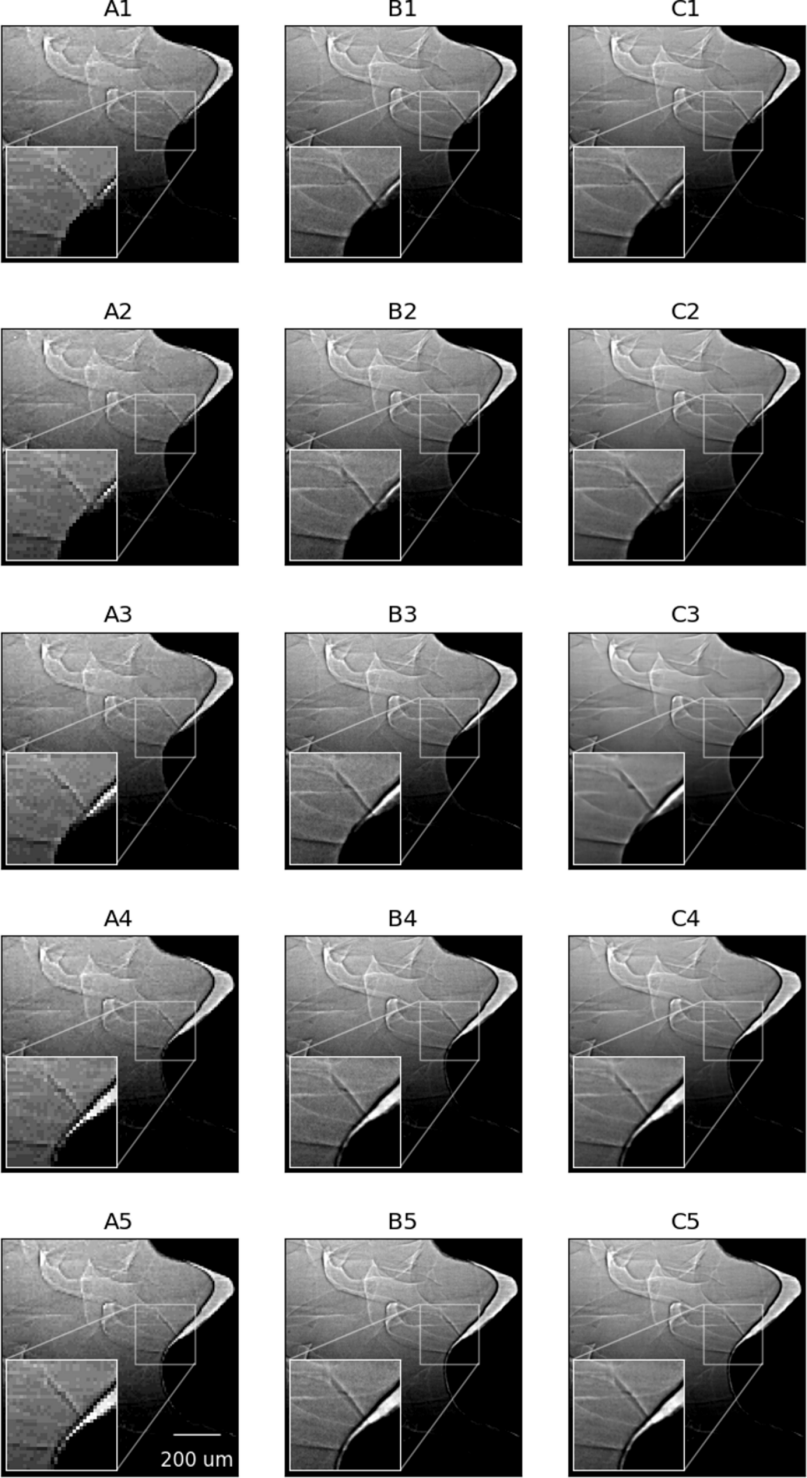
Selected image sequences of the LR images (column A), HR images (column B) and HR images reconstructed using EDVR-STF (column C) from Case 2. For each image sequence, images are shown from frames *i* − 10 (row 1), *i* − 9 (row 2), *i* (row 3), *i* + 9 (row 4) and *i* + 10 (row 5), respectively. To reconstruct HR images in the frame range [*i* − 10, *i* + 10], only HR images from frames *i* − 10 and *i* + 10 were used. No Poisson noise was generated for the testing data.

**Figure 5:**
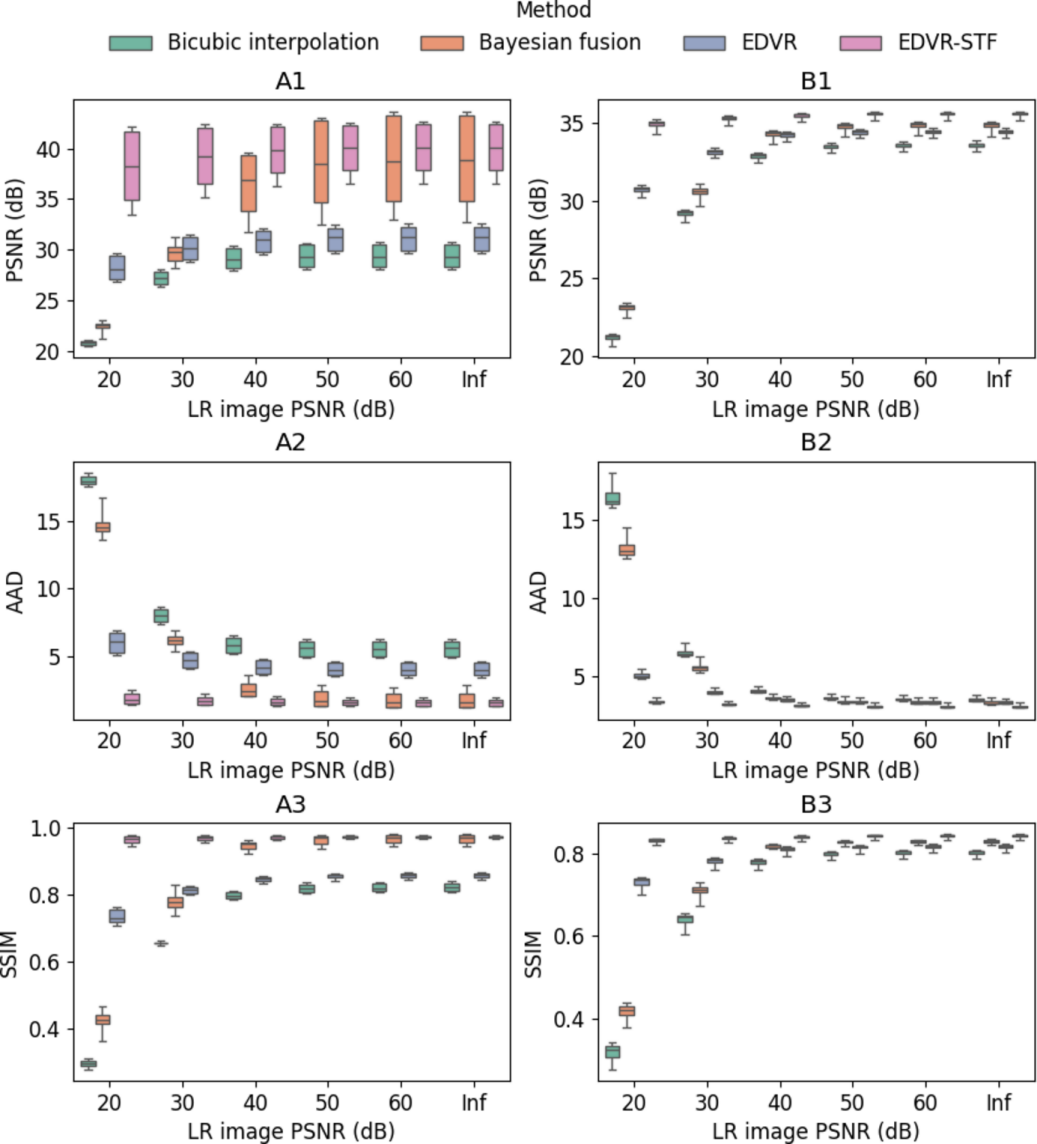
Reconstructed HR frame PSNR (in dB) (row 1), AAD (row 2) and SSIM (row 3), each as a function of the LR image PSNR that was used to generate the Poisson noise in the input LR frames, based on bicubic interpolation, Bayesian fusion, EDVR and EDVR-STF. Results were evaluated on Case 1 (column A) and Case 2 (column B) and are presented as box plots. Under each test condition, the same samples as used for EDVR-STF were used to test all three other algorithms.

**Figure 6:**
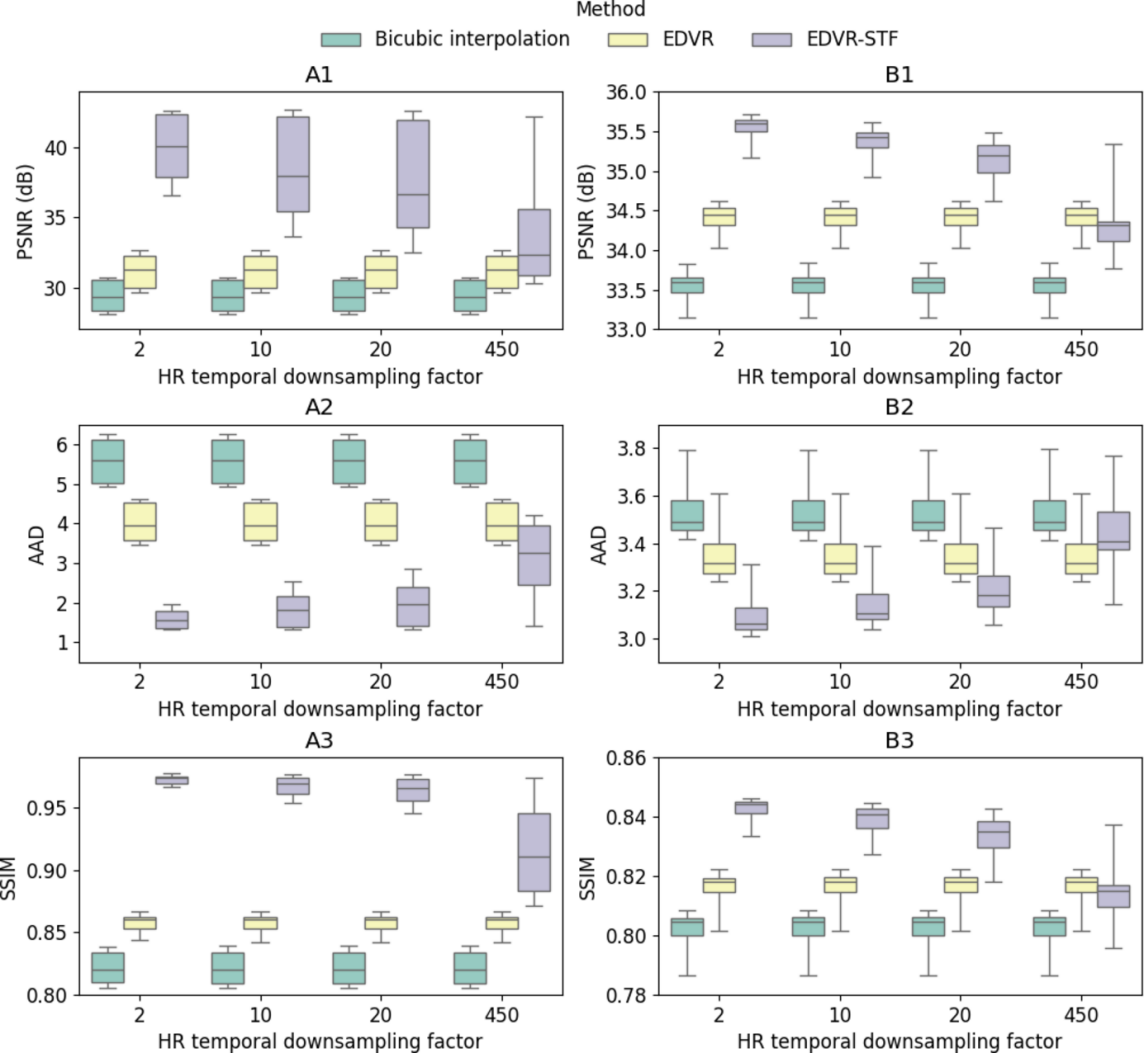
Reconstructed HR frame PSNR (in dB) (row 1), AAD (row 2) and SSIM (row 3), each as a function of the HR frame sequence down-sampling factor, based on bicubic interpolation, EDVR and EDVR-STF. Results were evaluated on Case 1 (column A) and Case 2 (column B) and are presented as box plots. The LR separation was one across all plots. Under each test condition, the same samples as used for EDVR-STF were used to test both other algorithms. No Poisson noise was generated for the testing data.

**Figure 7:**
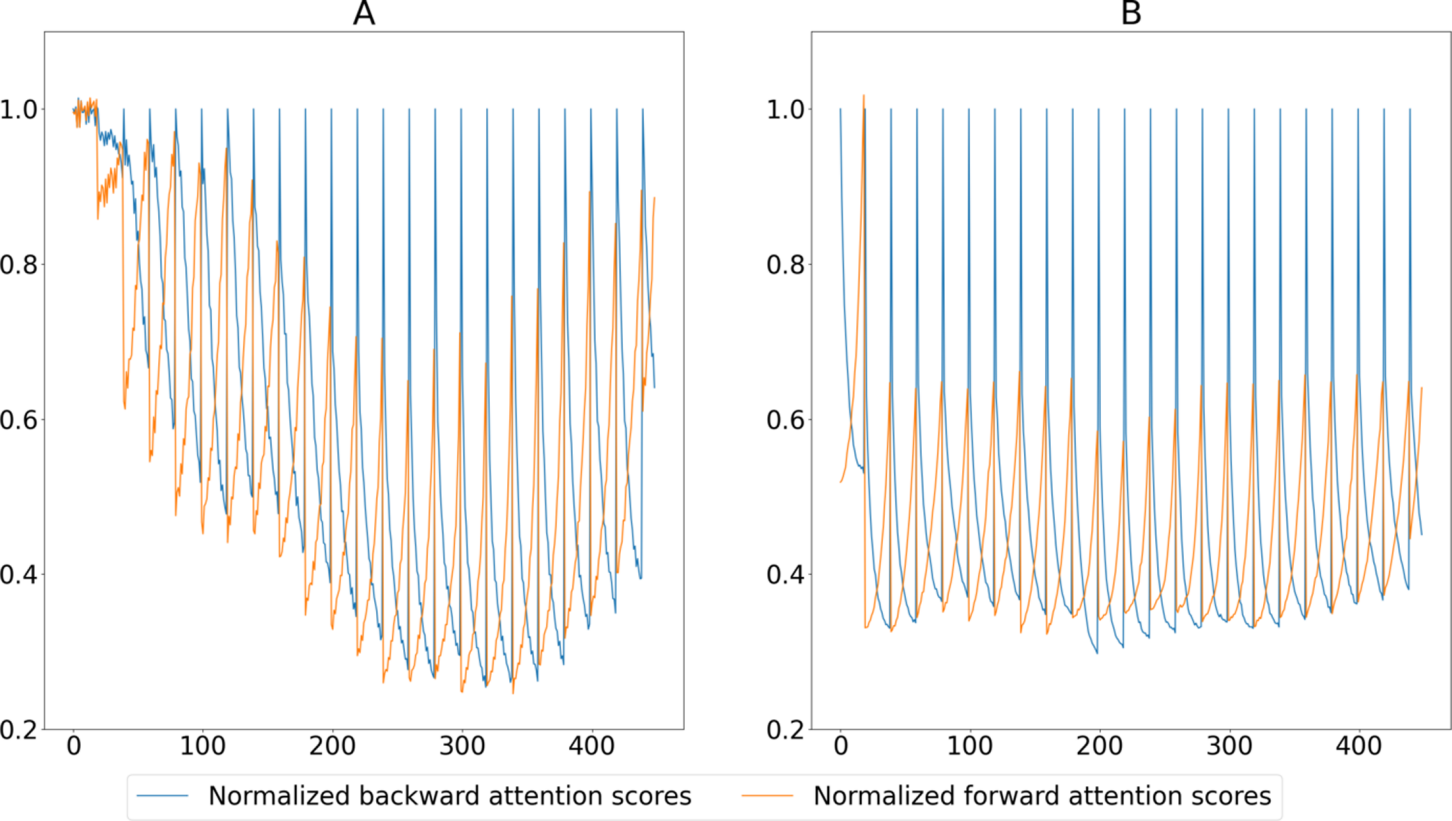
Normalized backward and forward attention scores of the EDVR-STF model to predict continuous target HR frames in Case 1 and Case 2. For both cases, the LR frame separation was set to one and the HR image sequence was down-sampled by a factor of 20. No Poisson noise was generated for the testing data.

**Figure 8:**
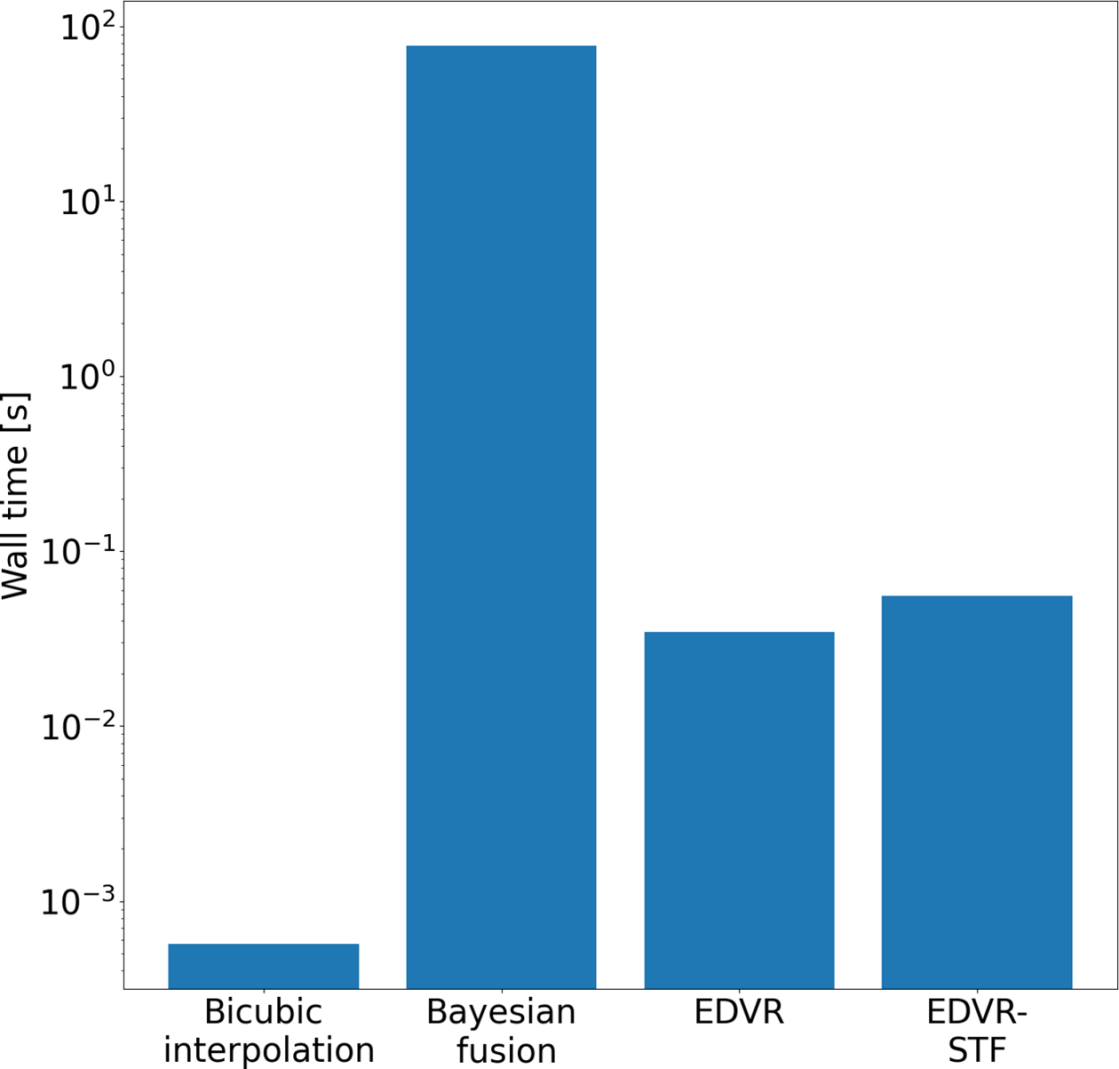
Wall times of the bicubic interpolation, Bayesian fusion framework, EDVR and EDVR-STF reported as the median over all 100 realizations.

In this section, we report qualitative and quantitative analyses of the proposed EDVR-STF image reconstruction approach as applied to the two X-ray image sequences described in Section 2 (paragraph 1) and Section S1.3 of the supporting information, *i.e.* in applications of additive manufacturing and friction stir welding, respectively. For the purpose of illustration, we first show temporally contiguous LR, HR and reconstructed HR images of each data set in one selected time window (with 20 consecutive frames) (Figs. 2 to 4) and over a longer time span (with 200–300 consecutive frames; see the two supplementary videos), and compare local features before and after the image reconstruction. We then compare the proposed approach with the other three approaches described in Section 2 (paragraph 2) and Section S1.4 of the supporting information, *i.e.* bicubic interpolation, Bayesian fusion and EDVR super-resolution, when the LR images are subjected to Poisson noise with varying levels of PSNR (Fig. 5) and when the underlying frame rates of both LR and HR images vary (Fig. 6, and Figs. S3 and S4). To interpret better the behavior of the proposed deep learning-based approach, we show the attention scores of the target image to each corresponding HR image input to the algorithm as described in Section S1.5 of the supporting information and illustrate a decreasing pattern as the target image gets farther from the input HR image (Fig. 7, and Fig. S5). Lastly, we make a brief characterization of the computation times of each method under their most accessible implementations (Fig. 8).

### 3.1. Qualitative assessments

Fig. 2 ▸ illustrates typical results of the HR image reconstruction from three subsequent LR images (one frame apart) and two fixed HR images (spaced by 20 LR frames) for the application of additive manufacturing (Case 1). In particular, the results shown in panels C2–C4 are from time points when the actual HR images are not available (*i.e.* B2–B4 are ‘unseen’ ground truth images held out at testing time), whereas C1 and C5 are from time points when the actual HR images are available (*i.e.* B1 and B5 as the preceding HR images in the reconstruction input). The normalized attention scores for C2, C3 and C4 are 0.69, 0.41 and 0.32 (backward attention) and 0.30, 0.39 and 0.70 (forward attention), respectively. Fig. 3 ▸ highlights moving features of the reconstructed image (in comparison with the original HR and LR images) in Fig. 2 ▸ with equal frame separation from both the preceding and succeeding HR images. When the motionless image content is masked out (by pixel-wise division with the preceding image, as is typical processing in the field of additive manufacturing), the reconstructed images show the moving particles and the keyhole features with significantly improved clarity compared with the LR image (A). The LR image sequence, the HR image sequence and the reconstructed HR image sequence in the longer time span are presented in the supporting video for data set 1.

Fig. 4 ▸ shows another case of HR image reconstruction by applying the same model and pre/post-processing pipeline as in Fig. 2 ▸ to a distinct type of X-ray image sequence capturing friction stir welding (Case 2) (Agiwal *et al.*, 2022 ▸). As can be seen from the images, the object motion captured in images from Fig. 4 ▸ is significantly slower than that from Fig. 2 ▸, and the degradation in image quality after a four times down-sampling is visually less pronounced. The normalized attention scores for panels C2–C4 are 0.62, 0.36 and 0.34 (backward attention) and 0.34, 0.38 and 0.65 (forward attention), respectively. In particular, fine-scale details of the original LR, original HR and reconstructed HR images in Fig. 4 ▸ are shown in the insets of the corresponding panels. Overall, the reconstructed images show a noticeable improvement in restoring image textures compared to the original LR image (column A). The LR image sequence, the HR image sequence and the reconstructed HR image sequence over the longer time span are presented in the supporting video for data set 2.

### 3.2. Performances with varying LR image PSNRs

Fig. 5 ▸ shows the PSNR, AAD and SSIM of the four reconstruction algorithms evaluated on the two testing data sets with varying LR image PSNRs due to Poisson noise. Overall, due to data augmentation of the LR frames with Poisson noise during the training stage, both EDVR and EDVR-STF show more steady performance across different noise levels of the testing data. When the noise level is high (*e.g.* 20 dB LR image PSNR), deep learning-based algorithms perform significantly better than conventional approaches, with higher PSNR and SSIM and lower AAD. As the LR image PSNR increases, the performance of the Bayesian fusion framework improves significantly, outperforming EDVR at LR image PSNRs of 40 dB and 50 dB in Case 1 and Case 2, respectively. At baseline with no Poisson noise generation in the testing data, the maximum PSNR of the Bayesian fusion framework is higher than EDVR-STF (43.66 dB versus 42.67 dB) in Case 1 and lower than EDVR-STF (35.06 dB versus 35.71 dB) in Case 2. The median PSNR of the Bayesian fusion framework is lower than that of EDVR-STF, consistently in both cases. At each noise level in both Case 1 and Case 2, the Bayesian fusion framework, EDVR and EDVR-STF perform significantly better than bicubic interpolation. The other two performance indices show consistent patterns among the four methods and as the blank scan factor changes.

### 3.3. Performances with varying image separations

Fig. 6 ▸ shows the PSNR, AAD and SSIM for bicubic interpolation, EDVR and EDVR-STF evaluated on the two testing data sets with varying down-sampling factors for temporal down-sampling of the HR image sequence and with the original frame separation of the LR image sequence. Overall, the performance of EDVR-STF decreases as the down-sampling factor increases. At the maximum down-sampling factor, where the HR frames are available only at the times when the first and last LR frames have been sampled, the PSNR of EDVR-STF is lower than that of EDVR in Case 2 (34.31 dB versus 34.45 dB) and higher than that of EDVR in Case 1 (32.34 dB versus 31.31 dB). However, EDVR-STF still outperforms all other algorithms in most situations considered, demonstrating the value of fusing HR video streams to boost the overall accuracy of the image sequence reconstruction. At each temporal down-sampling factor in both Case 1 and Case 2, both EDVR and EDVR-STF perform significantly better than bicubic interpolation. The other two performance indices show consistent patterns among the three methods and as the temporal down-sampling factor increases. In the supporting information (Figs. S3 and S4), the same results with larger frame separations (two and three, respectively) of the LR image sequence are shown. Consistent trends in all performance indices can be observed.

### 3.4. Normalized attention scores

Fig. 7 ▸ shows the normalized backward and forward attention scores obtained from the same two testing data sets. Between each pair of subsequent HR frames, the backward attention scores decrease as the target HR frame is far away from its preceding HR frame, and the forward attention scores increase accordingly. Fig. S5 further shows the temporal distributions of the normalized backward and forward attention scores in Case 1 as the frame number of the target HR frame changes in relation to its neighboring HR frames used for the reconstruction. In Fig. S5(*a*) when the target frame is close to its preceding HR frame (*e.g.* one frame after it), the corresponding attention scores are distributed primarily above 0.8. As the target frame is away from its preceding HR frame, a decreasing trend can be observed in the backward attention scores, which signifies the less important role the preceding HR frame plays in improving the resolution in the target frame. When the target frame number is close to that of its succeeding HR frame (*e.g.* 19 frames after the preceding frame), the majority of the attention scores are below 0.3. The opposite trend can be observed in the case of the forward attention scores, as illustrated in Fig. S5(*b*).

### 3.5. Computation times

Computation times for the four algorithms are shown in Fig. 8 ▸. The bicubic interpolation and Bayesian fusion framework both execute end to end, with wall times of 0.0006 s and 77.4044 s, respectively, on an Intel Xeon Gold 6334 CPU at 3.60 GHz to reconstruct an HR image with 400 × 1024 pixels. The EDVR and EDVR-STF models require a fixed time for model training. Once the models are trained, inference executes end to end with wall times of 0.0408 s and 0.0665 s, respectively, on one Nvidia A-100 SXM4 GPU (40 GB memory) to reconstruct an HR image with 400 × 1024 pixels. The bicubic interpolation was based on the *OpenCV* (https://opencv.org/) function resize. The Bayesian fusion algorithm was custom built following details given by Xue *et al.* (2017 ▸). The wall time of each method was estimated as the median wall time over 100 runs.

## 4. Discussion

In this paper, we have presented the first study that attempts to fuse two X-ray image sequences that are optimized for imaging speed and spatial resolution, respectively, and reconstructed the target image sequence that enjoys both high spatial resolution and high frame rate. In the current workflow of a UHS X-ray experiment, each individual high-speed camera is optimized to fulfill a dedicated role. However, given its optimal configuration, one single camera’s utility usually only covers a partial aspect of the entire envelope of the high-speed X-ray radiography experiment. In order to increase continually the scientific value that can be realized by the imaging experiment, the synergistic effect of multiplexing high-speed cameras must be better exploited. In this respect, a model that coordinates functionally distinct cameras and consolidates their respective imaging capabilities could greatly propel developments for the entire user community of UHS X-ray radiography. Due to the excellent performance of machine learning in modeling complex data structures as in the application of spatio-temporal analysis (Shi & Yeung, 2018 ▸), we concentrated on a deep convolutional neural network in this study, termed EDVR-STF.

In order to benchmark the proposed EDVR-STF model, we compared its performance with three other methods, namely the baseline EDVR model, the Bayesian fusion framework and the single-frame bicubic interpolation, based on three commonly used metrics, *i.e.* PSNR, AAD and SSIM. On one hand, the baseline EDVR model used the same LR images as input and was trained using the same configuration, differing from EDVR-STF only in the feature extraction branch to couple the HR feature maps. The purpose of including the baseline EDVR model was to understand the effect of mixing HR frames with LR frames on restoring the target HR frame. On the other hand, the Bayesian fusion framework used both HR and LR images as in EDVR-STF as input and was intended to provide a benchmark for the effect of utilizing a deep learning-based spatio-temporal fusion model. It should be noted that, in its current implementation, the Bayesian framework only learns pixel-level calibration models, which is in drastic contrast to the deep learning-based framework.

When the four image reconstruction approaches were compared, two variables were chosen, namely the frame separations of the HR image sequence and the Poisson noise level in the LR image sequence. In the evaluation of high-speed cameras, the frame rate is one of the most crucial specifications of the equipment, which could conveniently be used to stratify the instruments according to their distinct use scenarios. In this study, we concentrated on varying the frame separation of the HR image sequence, whereas the LR image sequence was intended to provide a well sampled time series of the target event. As a result, the configuration of the LR image sequence was contextualized within the specific scientific community utilizing the X-ray experiment. Poisson noise has been widely recognized as the dominant source of image noise under the majority of illumination conditions (Ikeuchi, 2021 ▸). In an X-ray experiment involving indirect detectors, the Poisson noise occurs with the random arrival of photons that get converted from the incoming X-ray beam, also termed ‘shot noise’. Due to its signal-dependent nature, shot noise often varies with the physical configurations of an imaging system, such as the exposure levels and sensor gains (Ikeuchi, 2021 ▸). In practice, the physical configurations of UHS cameras have often resulted in higher noise levels than high-speed cameras (Ren *et al.*, 2023 ▸). Therefore, in this study, Poisson noise was generated only in the LR images to demonstrate method robustness to shot noise as a proof of concept. Restoration of video quality in the presence of more complex types of process noise during the X-ray imaging experiment will be left for a future investigation. Testing image reconstruction algorithms on these configurations could thus allow efficient assessment of the feasibility of novel experimental protocols for high-speed X-ray radiography.

All reconstruction algorithms were tested on two independent data sets. From both data sets, EDVR-STF showed an overall higher performance than those of EDVR and the Bayesian fusion framework under each frame separation condition and each LR image PSNR condition, which in turn demonstrates the value of coupling neighboring HR image features with LR image features and of using a deep learning-based image reconstruction approach. On the other hand, the bicubic interpolation showed the lowest performance. Since the bicubic interpolation did not use any other frames than the LR reference frame itself, the results indicate the effective data association of all the other methods.

To allow a clear understanding of the performance of the proposed EDVR-STF model, we quantified the utilization of each of the two HR images as the input to reconstruct the target HR image. In particular, we derived numerical characteristics, termed the ‘forward attention score’ and ‘backward attention score’, from the attention map between each of the HR image’s feature maps and the reference LR feature maps. To reconstruct different target HR frames in an image sequence, the same set of input HR frames is not likely to play the same role. As a result, the availability of quantitative metrics could provide valuable insight into the model performance under different device settings and with complex temporal dynamics of the underlying event. Since feature fusion is only one module of the entire spatio-temporal fusion model, interpretation of abnormally low attention scores needs more caution towards complex confounding effects. Nonetheless, the attention scores can be used to indicate the internal states of the model at inference time and, along with the context information of the specific X-ray imaging experiments, identify unwanted working conditions of the equipment.

The proposed EDVR-STF model was intended to show preliminary results of applying a deep learning-based spatio-temporal fusion framework to solve the task of HR image sequence reconstruction. As a result, we concentrated on the specific configuration of fusing HR and LR image sequences with a fixed four times difference in each spatial dimension, while allowing the model performance to scale with variable frame rates that can be configured for the HR video. As the primary challenge of high-speed X-ray radiography is in the large difference in the camera frame rate, we expect the proposed model architecture to be able to handle the majority of scenarios from the user community. In practice, the proposed model could be combined with other super resolution/spatio-temporal fusion methods to enable variable spatial up-sampling factors around the fixed one. The proposed model training and inference could also be extended to support other tasks at a light source, such as improving the acquisition and reconstruction workflow of tomographic imaging data (Liu *et al.*, 2019 ▸; Liu *et al.*, 2020 ▸; Benmore *et al.*, 2022 ▸).

In this work, we pretrained the model on a diverse training data set outside the domain of X-ray imaging and transferred the model to a dedicated X-ray image set from the field of additive manufacturing. The effort to improve model generalizability further by curating a diverse training data set within the X-ray imaging modality, from an authentic dual-camera X-ray imaging setting, improving the model architecture and hence scalability with increasing training sample size, and creating more general imaging conditions at training time, will be left to be investigated in the future, with a foundation model trained on multiple image restoration tasks (Ma *et al.*, 2024 ▸).

## 5. Conclusions

In this paper, we have investigated the use of a deep learning-based spatio-temporal fusion algorithm to integrate two image sequences with high frame rate and high spatial resolution, respectively, and reconstruct target image sequences with high frame rate, high spatial resolution and high fidelity at the same time. The algorithm is implemented in Python and publicly available at https://github.com/xray-imaging/XFusion. With input image sequences of four times lower spatial resolution and 20 times lower frame rate, respectively, it has achieved an average PSNR of more than 35 dB based on our test set with interpretable performance metrics of the attention scores. We therefore conclude that the proposed framework could reconstruct the target X-ray image sequence with high fidelity, with realistic physical configurations of high-speed and ultra-high-speed cameras.

## 6. Related literature

The following references, not cited in the main body of the paper, have been cited in the supporting information: Lim *et al.* (2017 ▸); Nah *et al.* (2019 ▸); Peters *et al.* (2015 ▸).

## Supplementary Material

Video recording of data set 1. DOI: 10.1107/S1600577525000323/tv5068sup1.avi

Video recording of data set 2. DOI: 10.1107/S1600577525000323/tv5068sup2.avi

Additional details and extra figures. DOI: 10.1107/S1600577525000323/tv5068sup3.pdf
