# Supplementary material for: Deep learning-based spatio-temporal fusion for high-fidelity ultra-high-speed X-ray radiography
Source: J Synchrotron Radiat. 2025 Feb 12;32(Pt 2):432–41. doi: 10.1107/S1600577525000323 (PMC11892903; doi:10.1107/S1600577525000323)
Supplement: Supplementary file 3 [file s-32-00432-sup3.pdf]

JOURNAL OF
SYNCHROTRON
RADIATION

Volume 32 (2025)

Supporting information for article:

Deep learning-based spatio-temporal fusion for high-fidelity ultra-high-speed X-ray radiography

Songyuan Tang, Tekin Bicer, Tao Sun, Kamel Fezzaa and Samuel J. Clark

Deep learning-based spatio-temporal fusion for high-fidelity ultra-high-speed x-ray radiography

Supplementary Information

S1. Method

S1.1 Model architecture

As shown in Figure S*1*, the model consists of a cascade of four modules; namely, input feature extraction, feature alignment, feature fusion, and output image reconstruction. The input feature extraction module consists of independent and shared convolutional layers to process the LR and HR images separately and altogether. Each convolutional layer is followed by an activation layer to propagate extracted features between layers. 1 and 3 convolutional layers were included in the feature extraction modules for LR and HR images, respectively, and 5 residual blocks, each containing 2 convolutional layers with skip connections, were included in the subsequent shared feature extraction module. For the HR images, the last two convolution layers of their feature extraction module each was followed by an activation operation and a max pooling layer to reduce the resulting spatial dimensions of feature maps and keep them the same as those of the LR feature maps. A set of feature maps was thus output from the feature extraction module, corresponding to an input LR or HR frame at a distinct time point. The feature maps corresponding to the reference LR frame was identified as the "reference feature maps". The number of feature channels within each distinct set of feature maps was kept at 128 to balance the model complexity and the expressiveness of the learned feature maps.

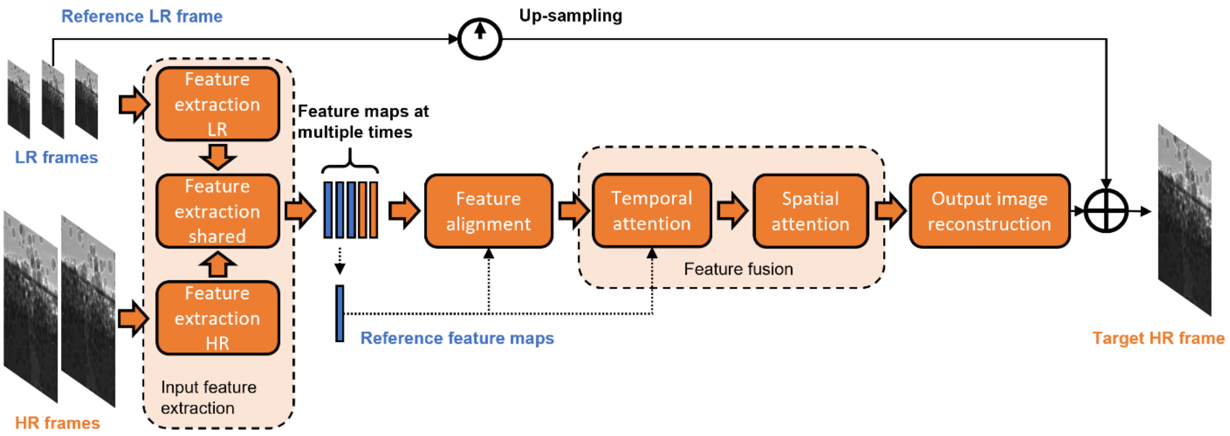

Figure S1. Architecture of the proposed spatio-temporal fusion model. Model inputs are consecutive LR and HR images that can be acquired from the corresponding high-speed and UHS cameras. Model output is a single HR image at the time when the reference LR image is acquired.

The subsequent feature alignment, feature fusion, and output image reconstruction modules were configured according to the original EDVR model architecture (Wang *et al.*, 2019). In particular, the feature alignment module used a pyramid of 3 levels, differing in the scale by a factor of 2 between subsequent levels in both spatial dimensions, to align each distinct set of feature maps with the reference feature maps in a coarse-to-fine manner. The feature fusion module consists of a temporal attention module followed by a spatial attention module to aggregate features unique to the HR images at the reference

time. More specifically, the temporal attention module was used to weight each set of aligned feature maps on a pixel-by-pixel manner based on their similarity to the reference feature maps and blend them in the reference time. The spatial attention module further learned with a large receptive field affine transformations to be applied to each pixel from each channel of the temporally fused feature maps (Tian *et al.*, 2020). The fused feature maps at the reference time were processed through the output image reconstruction module to approximate the residual image, which was added to the up-sampled reference LR image to restore the HR image at the same time point. In the reconstruction module, a cascade of 40 residual blocks, each containing 2 convolutional layers with skip connections were included, followed by 2 pixel shuffle up-sampling layers to recover the spatial dimension of the HR images (Lim *et al.*, 2017).

S1.2 Model training

A transfer learning strategy was adopted for model training to improve its generalization capability. In the pretraining stage, we used the realistic and diverse scenes (REDS) dataset of natural image sequences, which was an established benchmark on various video restoration tasks (Nah *et al.*, 2019). REDS contained 240 training videos, 100 frames per video, and 720×1280 pixels per frame. Each video was manually recorded to capture a diversity of objects and events. To reconcile the difference in the number of channels of the color images from REDS and the target x-ray images, we converted the color images to grayscale and established the pre-trained version of the EDVR-STF model that can be conveniently fine-tuned on x-ray data. In the fine-tuning stage, we used 547 videos recorded with a Photron FastCam SA-Z camera (Photron Inc., Japan) operated at a frame rate of 50 kHz during an operando high-speed synchrotron x-ray imaging experiment performed at the 32-ID beamline of the APS (Ren *et al.*, 2023).

Four videos were randomly sampled from all the videos and held out as validation data and the remaining were used as the training data. Each video contained 500 frames with 400×1024 pixels per frame. For the training data, random crops of 256×256 and 64×64 pixels were made on the corresponding HR and LR images to cover a spatially consistent region in each frame. Data augmentation included random horizontal flip, random vertical flip, and random rotation by 90 degrees, independently with a probability of 0.5. In addition, the frame separation between subsequent input LR frames was uniformly sampled at intervals 1, 2, and 3 at a time to increase the diversity of the input LR image sequence without significantly compromising the coherence among images. The frame separation between each of the two input HR images and the reference HR image was equal to that between subsequent input LR images scaled by a factor uniformly sampled from the discrete range of [-20,-1] (for the input HR image before the reference time) and [1,20] (for the input HR image after the reference time), respectively. The use of random frame separations among the input LR and HR was incorporated in an effort to desensitize the model from the underlying frame rate of the two video streams. Lastly, Poisson noise at each pixel of the LR images was simulated following (Wu *et al.*, 2020). The Poisson noise model characterizes the fluctuation in the quantity of photons as incident on the detectors. In particular, the blank scan factor b_0 was sampled in the range of 1 to 7 in the base-10 log scale (corresponding to a range of 10 to 10,000,000 in the linear scale) and kept constant for each pair of 3 LR images to equalize the resulting low-dose images based on their PSNR levels (approximately 10 dB to 70 dB). The introduction of Poisson noise during model training could effectively improve model robustness to varying image qualities, increasing the flexibility of the dual camera system with more diverse acquisition settings at the individual modular level. The model was then trained to minimize the Charbonnier loss (Wang *et al.*, 2019) for a total of 300,000 iterations with the Adam optimizer and an initial learning rate of 0.0001. The learning rate was updated following the cosine

annealing scheduler. The model was trained using PyTorch framework and Argonne Leadership Computing Facility (ALCF) resources.

S1.3 Data pre-processing

For the first video type in the testing data, a total of 450 continuous frames were used, with 400×1024 pixels per frame. For the second video type, a total of 450 continuous frames were used and each frame contained 1024×1024 pixels. The original frames within each video were kept as the HR frames and binning (Peters *et al.*, 2015) was applied to these frames to create another sequence of LR frames. For the resulting HR and LR image sequences, pixel values were clipped at the 0.35^{th} and 99.65^{th} percentiles according to their histograms, respectively, and the corresponding minimum and maximum pixel values across frames were in turn used to scale the pixel values of each individual frame following the min-max normalization rule. To further emulate different combinations of high-speed camera frame rates in a dual camera system, the frame separation between each LR image and its neighbouring LR images as inputs to the reconstruction algorithm was incremented from 1 to 3, and the original HR image sequence was resampled by keeping every 2nd (2× lower frame rate), 10th (10× lower frame rate), 20th (20× lower frame rate) frames, and ultimately only the leading and trailing frames (450× lower frame rate). To further emulate different shot noises the two cameras were subjected to, 5 distinct levels of the blank scan factor b_0 for each x-ray video, leading to PSNRs (relative to the LR frames) of approximately 20 dB to 60 dB, (in increments of 10 dB), were used to synthesize Poisson noise (details refer to the previous paragraph) in the LR image sequence.

S1.4 Baseline reconstruction methods

The Bayesian fusion framework used a similar input structure, i.e., 1 reference LR frame, 2 more LR frames and 2 more HR frames to estimate the HR frame at the same time as that of the reference LR frame. At every sampling time point, it first predicted the HR frame based on its temporal correlation with the two neighbouring HR frames and then updated it with the reference LR frame based on a generic image degradation model. By means of Gaussian models to represent the probabilistic change of pixel value across distinct spatial resolutions and in a time window, the proposed Bayesian framework led to a linear Kalman filter implementation to solve the overall fusion problem (Xue *et al.*, 2017). More specifically, the temporal change in each pixel value among subsequent frames was learned using a K-means clustering algorithm, with each cluster represented by a cluster mean vector and a covariance matrix and cluster assignment based on the Euclidean distance. Following (Xue *et al.*, 2017), each cluster's mean vector and covariance matrix were learned from the paired 3 LR frames across all positions sampled from the imaging plane. The number of clusters was determined as 5% of the number of pixels in each dimension of the LR image and geometrically averaged across both the height and width dimensions, based on a preliminary analysis presented in Figure S*2*. For short time series as in our proposed input structure (i.e., 3 LR frames), the number of clusters is expected to be small. Our experiments suggested the use of ∼10 clusters. Although this choice of cluster number may slightly over partition the time series feature space under the Euclidean distance metric, it could provide additional flexibility when dealing with complex dynamics in the underlying event.

The image degradation model was a linear model with linear interpolation of HR pixel values from non-overlapping square-shaped kernels and additive Gaussian noise (Xue *et al.*, 2017). HR and LR frames before and after the reference LR frame were each paired to calibrate the degradation model. Since the

Bayesian framework assumed the HR and LR frames to be obtained from the same time point, the corresponding frame separations were kept the same. For the baseline EDVR model, no HR frames were used during the inference, and the same 3 LR frames (one frame before, one frame at, and one frame after the reference time) were used to estimate the HR frame at the time of the reference LR frame. Analogous to the training procedure of EDVR-STF, it was pretrained on the same gray-scale REDS data set and then fine-tuned on the same x-ray images. The proposed EDVR-STF reconstruction algorithm was then tested, and the performance was compared with that of the bicubic interpolation, the Bayesian fusion, and the baseline EDVR super resolution model.

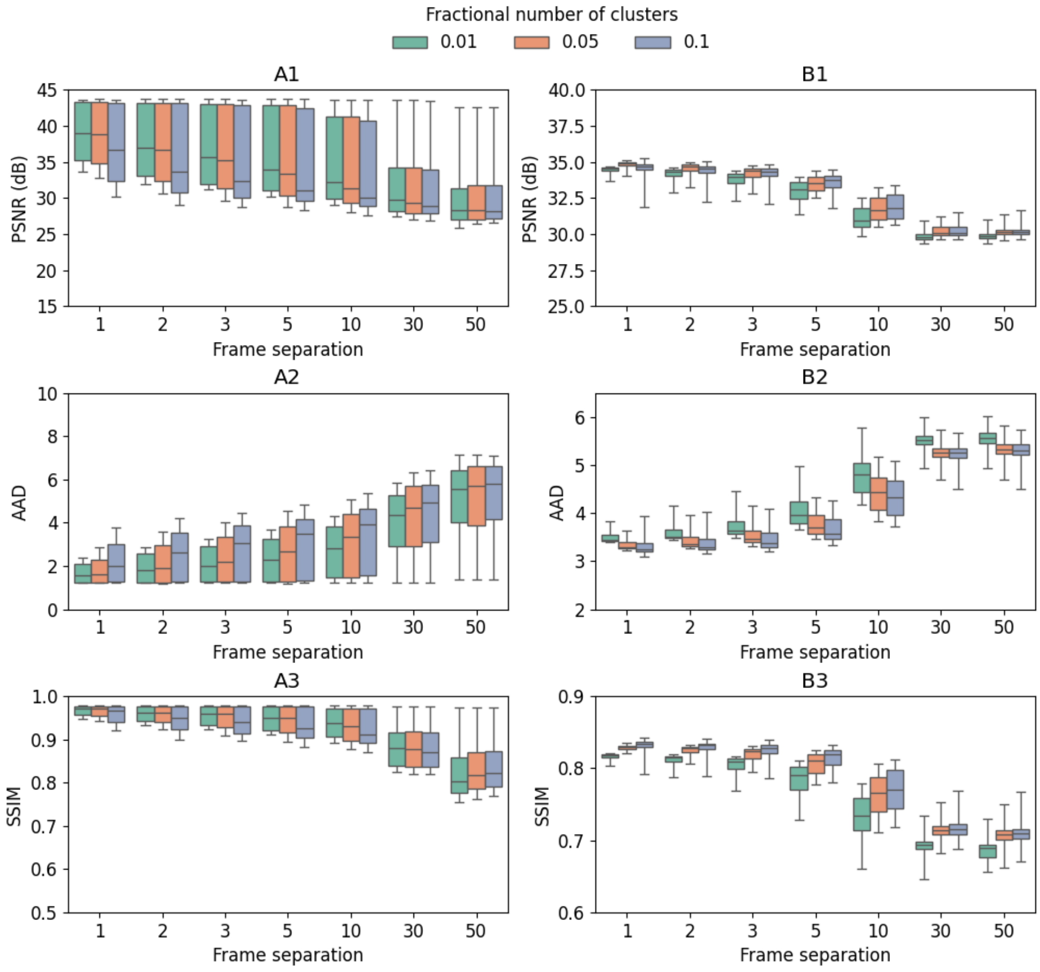

Figure S2. Reconstructed high resolution (HR) frame PSNR (dB) (row 1), AAD (row 2), and SSIM (row 3) each as a function of the cluster number fraction and the high/low resolution frame separation, based on Bayesian fusion. Results were evaluated on case 1 (column A) and case 2 (column B) of the main paper and presented as box plots. No Poisson noise was generated for the testing data.

S1.5 Attention scores

From the temporal attention module of the EDVR-STF model, the attention map between each set of feature maps corresponding to each of the two distinct HR input frames and the reference LR feature maps was spatially averaged. The resulting attention score characterizes the importance of each of the HR frames in restoring the reference LR frame. The attention score between each LR frame and the preceding and succeeding HR frames from the corresponding image sequences, termed as "backward attention score" and "forward attention score", respectively, were then each grouped by the preceding HR frame and normalized by the attention score between the HR and LR frames both acquired at the time of the preceding HR frame. The corresponding frame indices were also offset by that of the same preceding HR frame. Bivariate distributions of the locally normalized attention scores and their frame indices relative to the preceding HR frame were obtained in the form of two 2-D histograms, one for the backward attention scores and one for the forward attention scores. The normalized backward and forward attention scores provide a means of assessing the utilization of the corresponding HR images by the EDVR-STF model, hence objective quality assessment of the fusion, in the absence of the ground truth HR images as required by conventional quality indices.

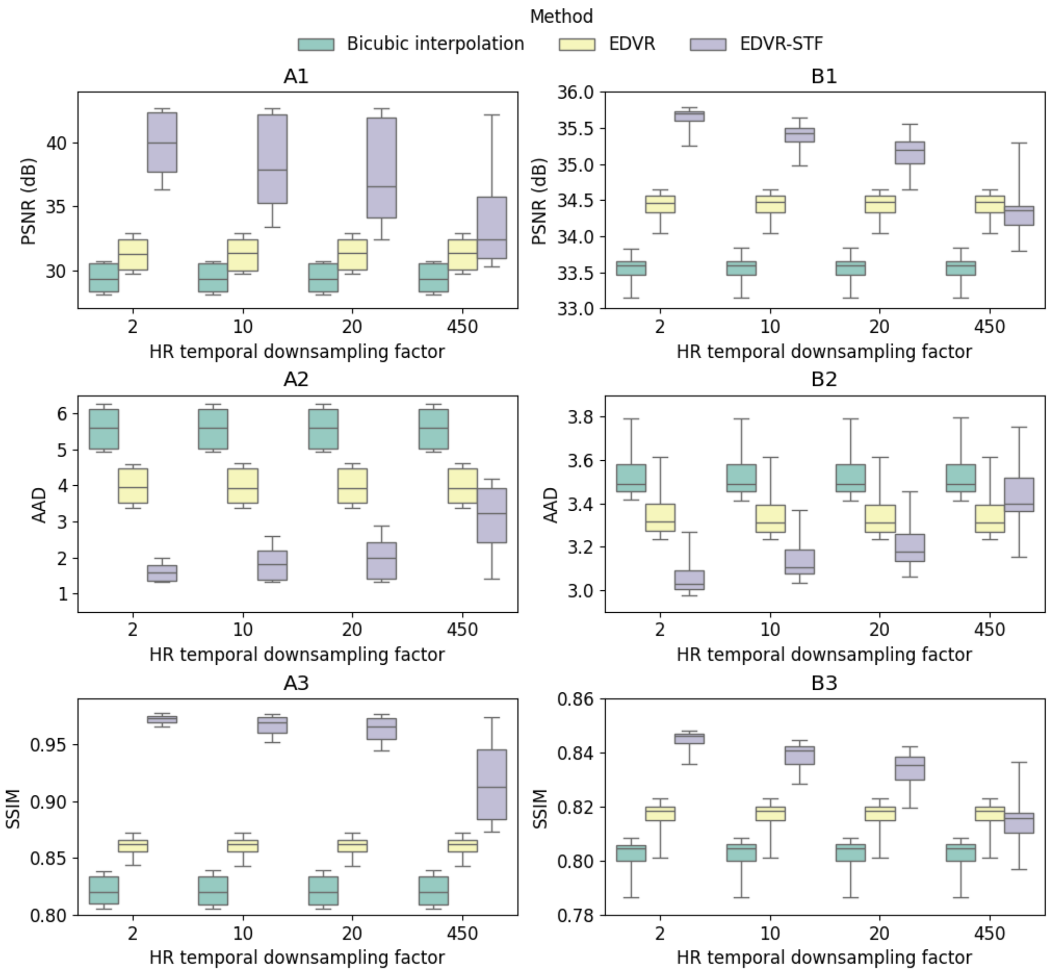

Figure S3. Reconstructed HR frame PSNR (dB) (row 1), AAD (row 2), and SSIM (row 3) each as a function of the HR frame sequence down-sampling factor, based on bicubic interpolation, EDVR, and EDVR-STF. Results were evaluated on case 1 (column A) and case 2 (column B) of the main paper and presented as box plots. LR separation was 2 across all plots. Under each testing condition, the same samples as used for EDVR-STF were used to test all other 2 algorithms. No Poisson noise was generated for the testing data.

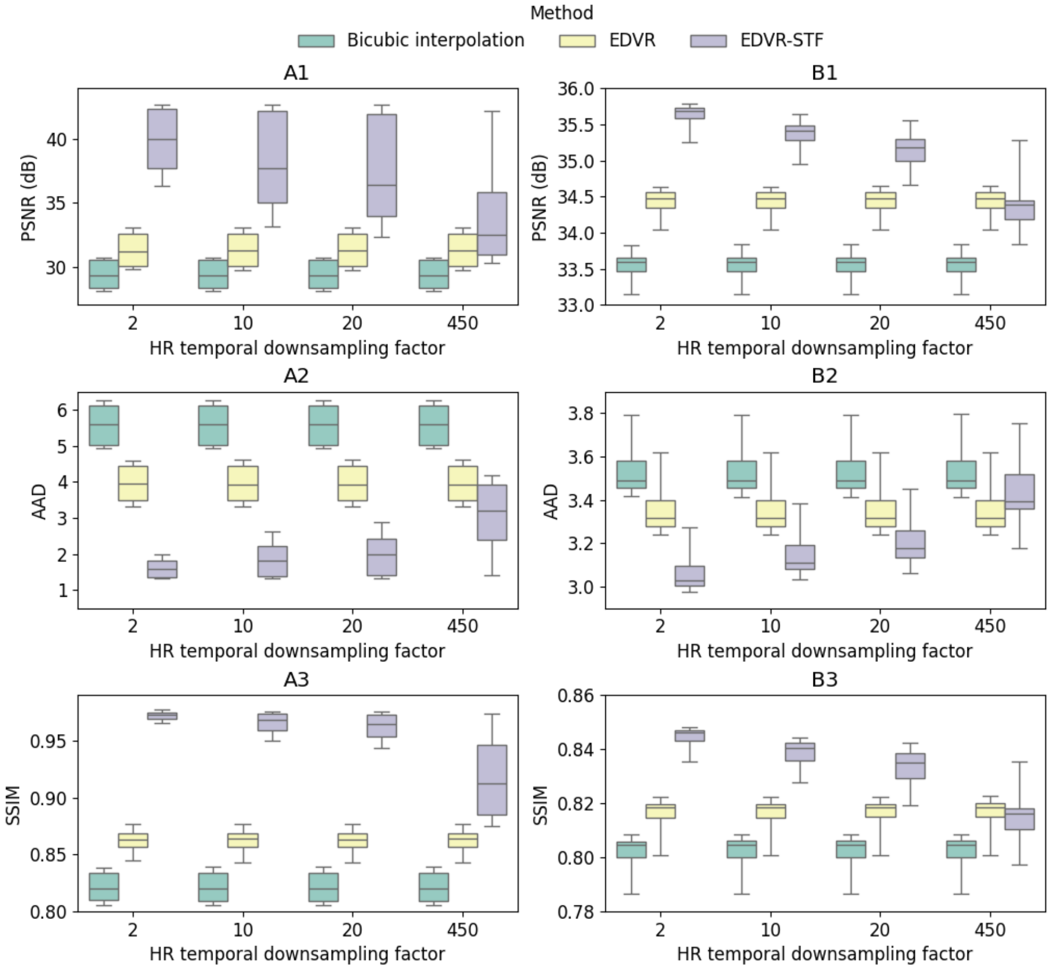

Figure S4. Reconstructed HR frame PSNR (dB) (row 1), AAD (row 2), and SSIM (row 3) each as a function of the HR frame sequence down-sampling factor, based on bicubic interpolation, EDVR, and EDVR-STF. Results were evaluated on case 1 (column A) and column 2 (column B) of the main paper and presented as box plots. LR separation was 3 across all plots. Under each testing condition, the same samples as used for EDVR-STF were used to test all other 2 algorithms. No Poisson noise was generated for the testing data.

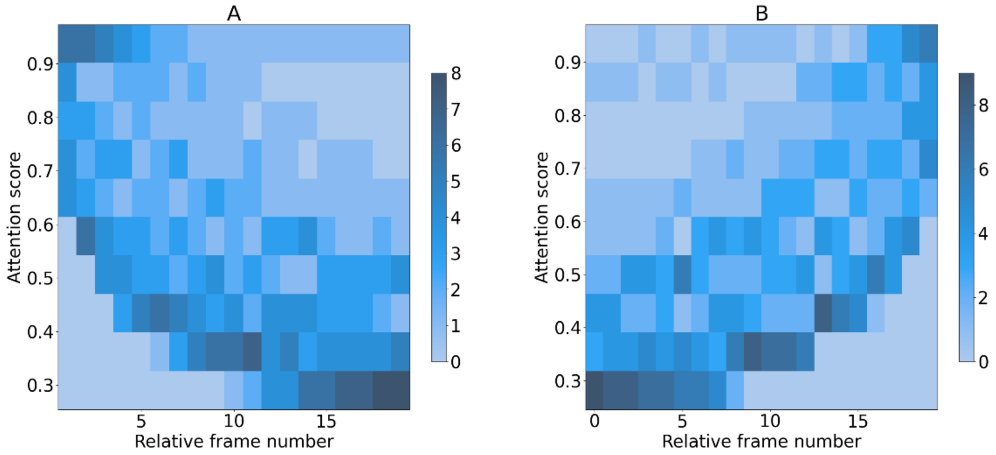

Figure S5. 2-D histograms of the normalized backward attention scores (A) and forward attention scores (B) and the relative frame numbers when the EDVR-STF model was evaluated on continuous frames from one testing data set (case 1). In the illustrated case, the LR frame separation was set to 1 and the HR image sequence was down-sampled by a factor of 20. No Poisson noise was generated for the testing data.

References

Lim, B., Son, S., Kim, H., Nah, S. & Mu Lee, K. (2017). *Proceedings of the IEEE conference on computer vision and pattern recognition workshops*, pp. 136-144.

Nah, S., Baik, S., Hong, S., Moon, G., Son, S., Timofte, R. & Mu Lee, K. (2019). *Proceedings of the IEEE/CVF conference on computer vision and pattern recognition workshops*, pp. 0-0.

Peters, C. J., Danehy, P. M., Bathel, B. F., Jiang, N., Calvert, N. & Miles, R. B. (2015). *31st AIAA Aerodynamic Measurement Technology and Ground Testing Conference*, p. 2565.

Ren, Z., Gao, L., Clark, S. J., Fezzaa, K., Shevchenko, P., Choi, A., Everhart, W., Rollett, A. D., Chen, L. & Sun, T. (2023). *Science* **379**, 89-94.

Tian, Y., Zhang, Y., Fu, Y. & Xu, C. (2020). *Proceedings of the IEEE/CVF conference on computer vision and pattern recognition*, pp. 3360-3369.

Wang, X., Chan, K. C., Yu, K., Dong, C. & Change Loy, C. (2019). *Proceedings of the IEEE/CVF conference on computer vision and pattern recognition workshops*, pp. 0-0.

Wu, Z., Bicer, T., Liu, Z., De Andrade, V., Zhu, Y. & Foster, I. T. (2020). *2020 IEEE/ACM Workshop on Machine Learning in High Performance Computing Environments (MLHPC) and Workshop on Artificial Intelligence and Machine Learning for Scientific Applications (AI4S)*, pp. 88-95. IEEE.

Xue, J., Leung, Y. & Fung, T. (2017). *Remote Sensing* **9**, 1310.
